# Integrated proteomics spotlight the proteasome as a therapeutic vulnerability in embryonal tumors with multilayered rosettes

**DOI:** 10.1093/neuonc/noad265

**Published:** 2023-12-30

**Authors:** Matthias Dottermusch, Ali Biabani, Tasja Lempertz, Yannis Schumann, Jelena Navolic, Shweta Godbole, Denise Obrecht, Stephan Frank, Mario M Dorostkar, Hannah Voß, Hartmut Schlüter, Stefan Rutkowski, Ulrich Schüller, Julia E Neumann

**Affiliations:** Center for Molecular Neurobiology (ZMNH), University Medical Center Hamburg-Eppendorf, Hamburg, Germany; Institute of Neuropathology, University Medical Center Hamburg-Eppendorf, Hamburg, Germany; Section of Mass Spectrometric Proteomics, University Medical Center Hamburg-Eppendorf, Hamburg, Germany; Center for Molecular Neurobiology (ZMNH), University Medical Center Hamburg-Eppendorf, Hamburg, Germany; Chair for High Performance Computing, Helmut-Schmidt University, Hamburg, Germany; Center for Molecular Neurobiology (ZMNH), University Medical Center Hamburg-Eppendorf, Hamburg, Germany; Center for Molecular Neurobiology (ZMNH), University Medical Center Hamburg-Eppendorf, Hamburg, Germany; Department of Pediatric Hematology and Oncology, University Medical Center Hamburg-Eppendorf, Hamburg, Germany; Division of Neuropathology, Institute of Medical Genetics and Pathology, University Hospital Basel, University of Basel, Basel, Switzerland; Center for Neuropathology and Prion Research, Ludwig Maximilian University, Munich, Germany; Section of Mass Spectrometric Proteomics, University Medical Center Hamburg-Eppendorf, Hamburg, Germany; Section of Mass Spectrometric Proteomics, University Medical Center Hamburg-Eppendorf, Hamburg, Germany; Department of Pediatric Hematology and Oncology, University Medical Center Hamburg-Eppendorf, Hamburg, Germany; Institute of Neuropathology, University Medical Center Hamburg-Eppendorf, Hamburg, Germany; Department of Pediatric Hematology and Oncology, University Medical Center Hamburg-Eppendorf, Hamburg, Germany; Children’s Cancer Research Center Hamburg, Hamburg, Germany; Center for Molecular Neurobiology (ZMNH), University Medical Center Hamburg-Eppendorf, Hamburg, Germany; Institute of Neuropathology, University Medical Center Hamburg-Eppendorf, Hamburg, Germany

**Keywords:** DNA methylation, embryonal tumor with multilayered rosettes, proteasome, proteome, transcriptome

## Abstract

**Background:**

Embryonal tumors with multilayered rosettes (ETMR) are rare malignant embryonal brain tumors. The prognosis of ETMR is poor and novel therapeutic approaches are desperately needed. Comprehension of ETMR tumor biology is currently based on only few previous molecular studies, which mainly focused on the analyses of nucleic acids. In this study, we explored integrated ETMR proteomics.

**Methods:**

Using mass spectrometry, proteome data were acquired from 16 ETMR and the ETMR cell line BT183. Proteome data were integrated with case-matched global DNA methylation data, publicly available transcriptome data, and proteome data of further embryonal and pediatric brain tumors.

**Results:**

Proteome-based cluster analyses grouped ETMR samples according to histomorphology, separating neuropil-rich tumors with neuronal signatures from primitive tumors with signatures relating to stemness and chromosome organization. Integrated proteomics showcased that ETMR and BT183 cells harbor proteasome regulatory proteins in abundance, implicating their strong dependency on the proteasome machinery to safeguard proteostasis. Indeed, in vitro assays using BT183 highlighted that ETMR tumor cells are highly vulnerable toward treatment with the CNS penetrant proteasome inhibitor Marizomib.

**Conclusions:**

In summary, histomorphology stipulates the proteome signatures of ETMR, and proteasome regulatory proteins are pervasively abundant in these tumors. As validated in vitro, proteasome inhibition poses a promising therapeutic option in ETMR.

**Key Points**

- Integrated proteomics provide a comprehensive view of molecular features of ETMR.
- Proteasome regulatory proteins are highly abundant in ETMR.
- The CNS-penetrant proteasome inhibitor Marizomib has potent cytotoxic effects on ETMR cells.

**Importance of the Study**

Despite recent advances in the molecular characterization of embryonal tumors with multilayered rosettes (ETMR), the prognosis for these rare pediatric brain tumors remains extremely poor. Proteomics have not been studied in ETMR yet, but hold the prospect to closely reflect functionally relevant tumor features. We performed integrated proteome analyses to provide a comprehensive view on novel as well as highly conserved molecular features of ETMR. Our investigations revealed an abundance of proteasome regulatory proteins in ETMR and demonstrated the therapeutic potential of proteasome inhibition in vitro. Our findings provide valuable insight into ETMR molecular biology and pave the way for novel therapeutic strategies.

Embryonal tumors with multilayered rosettes (ETMR) are rare and highly malignant brain tumors, which predominantly affect infants.^1^ ETMR can be categorized into three histological variants: embryonal tumor with abundant neuropil and true rosettes (ETANTR), ependymoblastoma (EBL), or medulloepithelioma (MEPL). ETANTR feature vast areas of neuropil intermixed with rosette-forming and dense clusters of primitive, undifferentiated cells. EBL predominantly display dense sheets of primitive cells forming multilayered rosettes. MEPL morphology is epithelioid and reminiscent of the primitive neural tube. Of note, morphological features tend to overlap between the different histological variants and ETMR may also shift between variants upon relapse.^2–4^ ETANTR, EBL, and MEPL do not significantly differ in their epigenomic profiles or clinical presentations, which suggests that they have a common origin and share core biological features.^5,6^

ETMR are molecularly characterized by distinct DNA methylation signatures and dysregulated expression of oncogenic miRNAs.^2,3^ Amplifications of 19q13.42 encoding the primate-specific miRNA cluster C19MC are the most frequent recurrent genomic alterations in ETMR.^5,7,8^ Remaining cases often harbor mutations affecting DICER1, a ribonuclease involved in miRNA processing.^2,9,10^ Recently, dysregulated miRNA processing has been linked to R-loop-associated chromosomal instability and upregulated DNA repair mechanisms in ETMR.^2,6^

As of today, the prognosis for ETMR remains extremely poor with a median time to death of under 12 months despite intensive treatment efforts.^7,11,12^ Further advancements in the molecular characterization of ETMR are urgently needed to improve current treatment regimens and develop novel therapeutic strategies. Proteomics has not been studied in ETMR yet, but holds out the prospect to reflect functionally relevant tumor features more closely. In this study we aimed to explore the proteome landscape of a case series of molecularly confirmed ETMR and the ETMR cell line BT183. We integrated proteome, transcriptome, and methylome data of embryonal brain tumors to provide an extended perspective on molecular features of ETMR and identify novel therapeutic vulnerabilities.

## Methods

### Human Tissue

Formalin-fixed, paraffin-embedded (FFPE) human tumor tissue of ETMR, atypical teratoid/rhabdoid tumors (AT/RT) and medulloblastoma (MB) were acquired from the Institute of Neuropathology, University Medical Center Hamburg-Eppendorf (UKE), Hamburg, the Center for Neuropathology, Ludwig Maximilians University (LMU), Munich, and the Division of Neuropathology, Basel University Hospital. Ethics approval was waived by the Ethics Committee of the Hamburg Chamber of Physicians (PV6007). The use of all tissue specimens for research upon anonymization was in accordance with local and national ethical standards and with the 1964 Helsinki Declaration and its later amendments.

### Histology and Immunohistochemistry

Tissue samples were fixed in 4% buffered formaldehyde, dehydrated, embedded in paraffin, and sectioned at 2 µm. H&E staining was performed according to standard laboratory protocols. Immunohistochemical stainings were performed on a Ventana BenchMark XT system (Roche Diagnostics). The following primary antibodies were used: SALL4 (ab57577, abcam, 1:50), Ki67 (SP6, Cell Marque, 1:750), SOX2 (ab97959, abcam, 1:1000), MAP2C (M4403, Sigma–Aldrich, 1:3000), CD56 (MSK006, Zytomed, 1:2000), Synaptophysin (M7315, Dako, 1:500). As a chromogen, 3,3ʹ-diaminobenzine (DAB) was used.

### Sample Preparation for Mass Spectrometry

Punch biopsies and/or microdissected tissue sections were collected from FFPE embryonal brain tumors. For whole sample analyses, care was taken to compile samples, which sufficiently portray the histomorphology of the entire tumor. For paraffin removal, samples were incubated in 0.5 ml n-heptane at room temperature for 30 min, using a ThermoMixer (ThermoMixer 5436, Eppendorf). Samples were centrifuged at 14 000 g for 5 min and the supernatant was discarded. Samples were reconditioned with 70% ethanol and centrifuged at 14 000 g for 5 min. The supernatant was discarded. The procedure was repeated twice. Deparaffinized tissue samples and fresh frozen cell pellets were dissolved in 150 µL 1% w/v sodium deoxycholate (SDC) in 0.1 M triethylammonium bicarbonate buffer (TEAB) and incubated for 1 h at 95 °C for reverse formalin fixation. Samples were sonicated for 5 s at an energy of 25% to destroy interfering DNA. A bicinchoninic acid (BCA) assay was performed (Pierce™ BCA Protein Assay Kit, Thermo Scientific) to determine the protein concentration, following the manufacturer’s instructions. Tryptic digestion was performed for 20 μg protein, using the single-pot, solid-phase-enhanced sample preparation (SP3) protocol, as described by Hughes et al.^13^ Eluted peptides were dried in a Savant SpeedVac Vacuum Concentrator (Thermo Fisher Scientific) and stored at −20 °C until further use. Directly prior to measurement dried peptides were resolved in 0.1% FA to a final concentration of 1 μg/μL. In total 1 μg was subjected to mass spectrometry analysis.

### Mass Spectrometry Analyses

Liquid chromatography–tandem mass spectrometer (LC–MS/MS) measurements were performed on a quadrupole-ion-trap-orbitrap mass spectrometer (MS, QExactive, Thermo Fisher Scientific) coupled to a nano-UPLC (Dionex Ultimate 3000 UPLC system, Thermo Fisher Scientific). Tryptic peptides were injected into the LC system via an autosampler, purified, and desalted by using a reversed-phase trapping column (Acclaim PepMap 100 C18 trap; 100 μm × 2 cm, 100 A pore size, 5 μm particle size; Thermo Fisher Scientific), and thereafter separated with a reversed-phase column (Acclaim PepMap 100 C18; 75 μm × 25 cm, 100 A pore size, 2 μm particle size, Thermo Fisher Scientific). Trapping was performed for 5 min at a flow rate of 5 µL/min with 98% solvent A (0.1% FA) and 2% solvent B (0.1% FA in ACN). Separation and elution of peptides were achieved by a linear gradient from 2% to 30% solvent B in 65 min at a flow rate of 0.3 µL/min. Eluting peptides were ionized by using a nano-electrospray ionization source (nano-ESI) with a spray voltage of 1800 V, transferred into the MS, and analyzed in data-dependent acquisition (DDA) mode. For each MS1 scan, ions were accumulated for a maximum of 240 ms or until a charge density of 1 × 1^6 ions (AGC target) was reached. Fourier-transformation-based mass analysis of the data from the orbitrap mass analyzer was performed by covering a mass range of 400—1200 *m*/*z* with a resolution of 70 000 at *m*/*z* = 200. Peptides with charge states between 2+ and 5 + above an intensity threshold of 5000 were isolated within a 2.0 *m*/*z* isolation window in top-speed mode for 3 s from each precursor scan and fragmented with a normalized collision energy of 25 %, using higher energy collisional dissociation (HCD). MS2 scanning was performed, using an orbitrap mass analyzer, with a starting mass of 100 *m*/*z* at an orbitrap resolution of 17 500 at *m*/*z* = 200 and accumulated for 50 ms or to an AGC target of 1 × 10^5. Already fragmented peptides were excluded for 20 s.

### Acquisition and Preprocessing of Proteome Data

LC–MS/MS raw spectra were searched against a reviewed human Swissprot database, obtained in February 2022, containing 20 300 entries, using the SEQUEST algorithm integrated into the Proteome Discoverer software (v 3.0, Thermo Fisher Scientific). A maximum number of 2 missing tryptic cleavages was set. Peptides between 6 and 144 amino acids were considered. Carbamidomethylation was set as a fixed modification for cysteine residues and the oxidation of methionine, and pyro-glutamate formation at glutamine residues at the peptide N-terminus, as well as acetylation of the protein N-terminus was allowed as variable modifications. A strict cutoff (false discovery rate (FDR) < 0.01) was set for peptide and protein identification. Protein quantification was carried out, using the Minora Algorithm, implemented in Proteome Discoverer. For quantification, only unique peptides were used. Retrieved protein abundancies were normalized at the protein level, using all proteins quantified. Scaling was disabled. Protein abundances were log2 transformed and normalized using sample-wise median centering.

### Acquisition of Transcriptome Data

Gene expression data of embryonal brain tumor samples (CEL files) were acquired from the publicly available data sets GSE10327,^14^ GSE122077,^6^ GSE70678,^15^ and GSE73038.^16^ Sample annotations were obtained from the respective GEO deposits and the corresponding publications. Sample annotations were checked for redundancy. Potential duplicate samples were excluded from the analyses. Sample files were simultaneously processed using Affymetrix TAC4.0 software with default parameters (RNA normalization). Transcripts were collapsed using GSEA software v4.0.3 of the Broad Institute.^17^

### Acquisition and Preprocessing of Methylome Data

DNA was isolated using the ReliaPrep™ FFPE gDNA Miniprep System (Promega) according to the manufacturer’s instructions. Approximately 100–500 ng DNA was used for bisulfite conversion by the EZ DNA Methylation Kit (Zymo Research). Afterward, the DNA Clean & Concentrator-5 (Zymo Research) and the Infinium HD FFPE DNA Restore Kit (Illumina) were used to clean and restore the converted DNA. The Illumina Infinium Methylation EPIC BeadChip Kit was used to quantify the methylation status of CpG sites on an Illumina iScan system. Raw methylation array data (idat files) were processed using the minfi package^18^ in R software.^19^ Stratified quantile normalization preprocessing was performed. Probes on sex chromosomes, probes with a detection *P*-value of or above 0.01, probes with SNPs at the CpG site, and cross-reactive probes were excluded from the analysis.

### Gene Set Enrichment Analysis (GSEA)

For GSEA of proteome and transcriptome data, proteins and RNAs were ranked according to the product of fold-change and negative log10-transformed *P*-values comparing ETMR with AT/RT and MB. To obtain histomorphology-independent ranks, the ranking was performed separately for EBL/MEPL and ETANTR cases and the ranks were subsequently combined prior to performing GSEA. Gene set size ranges were set from 10 to 250 for proteins and from 10 to 600 for RNA. All *P*-values derived from GSEA were BH-adjusted and considered significant with a *P*-value ≤ .05.

### GO Over-Representation Analysis (ORA)

For ORA of proteome data, significant differential protein abundance was determined by Welch’s *t*-test. *P*-values ≤ .05 were considered significant. ORA was performed by testing for GO enrichment of significant proteins within the universe of all proteins represented in the data. Gene set size ranges were set to 10–250 for proteins. GO terms with *P*-values lower than 0.05 and protein counts higher than 4 were considered over-represented. GO terms with a Resnik’s and Lin’s similarity measure over 0.4 were considered semantically similar. For simplification of category-gene-net (CNET) plots, terms with semantic redundancy to terms with higher significance were removed. For simplification of Venn diagrams, non-overlapping terms with semantic redundancy to overlapping terms were removed.

For ORA of methylome data, promotor-associated CpG methylation, and protein levels were investigated for sample-wise Spearman correlation across embryonal brain tumors. Promotor-association was defined as TSS200, TSS1500, or 5ʹUTR according to the ChAMP package (version: 2.24.0).^20^ Proteins were considered to correlate significantly with methylation levels when at least one of the gene promotor-associated CpG demonstrated a Benjamini, Hochberg, and Yekutieli (BY)-adjusted *P*-value ≤ .05. ORA was performed on all significantly correlating proteins within the universe of all significantly differentially abundant proteins in ETMR. Gene set size ranges were set to 5–150. GO terms were considered over-represented when at least 2 and more than 10% of gene set members showed a significant correlation between protein and methylation levels.

### Proteome Data Set Integration

Processed proteome data of pediatric brain tumors was obtained from publicly available data sets (PDC000180,^21^ accessed via https://pdc.cancer.gov). Batch effects between the individual TMT plexes were corrected using ComBat.^22^ For integration with our case series, the medians of all samples were shifted to 0 and the data was scaled to a median absolute deviation of 1 per sample. COCONUT^23^ was deployed to correct batch effects, using medulloblastomas of the same molecular subtypes (2 SHH-MH, 2 Gr4-MB, 1 WNT-MB) as references between the datasets. The reference samples were excluded in further analyses. Batch effects between proteome data of in-house cell line samples and in-house embryonal brain tumor tissue samples were corrected using ComBat.^22^

### Cell Culture

BT183 cells were cultured in NeuroCult NS-A Proliferation Kit (Human), supplemented with heparin (2 µg/ml), epidermal growth factor (EGF; 20 ng/mL), and fibroblast growth factor (FGF; 20 ng/ml) (STEMCELL Technologies). BT16 were grown in Roswell Park Memorial Institute medium (RPMI 1640 (+l-Glutamine)), including 15% fetal bovine serum (FBS) and 25 mM HEPES buffer (Gibco, Thermo Fisher Scientific). D283 were grown in Eagle’s Minimum Essential Medium (EMEM) (ATCC) including 10% FBS. Media were additionally supplemented with 100 U/ml penicillin/streptomycin (Gibco, Thermo Fisher Scientific). All cell line identities were confirmed via cell line authentication services (Eurofins Genomics) and/or global DNA methylation profiling.

### Cell Viability Assays

Cells were seeded onto white 96-well plates and incubated for 16 h. Marizomib (SML1916, Sigma-Aldrich) was added at different concentrations in 6 technical replicates. After 72 h, cell viability was measured using the CellTiter-Glo reagent (Promega) according to the manufacturer’s instructions. Luminescence was measured using an automated plate reader (GloMax Discover microplate reader). Dose–response curves and half-maximal inhibitory concentration (IC_50_) values were calculated with GraphPad Prism software v8.4.3.

### Data and Image Processing

Computational analyses were performed using the R software.^19^ GSEA and ORA analyses were performed using the clusterProfiler package (version: 4.2.2).^24^ Single sample GSEA (ssGSEA) was performed using the GSVA package (version: 1.42.0).^25^ Non-negative matrix factorization was performed using the NMF package (version: 0.26)^26^ on processed and scaled log2 transformed protein data. Proteins were searched for potential targets with the use of QIAGEN IPA (QIAGEN Inc., https://digitalinsights.qiagen.com/IPA).^27^ Tissue slides were digitalized with a Hamamatsu NanoZoomer 2.0-HT C9600 whole slide scanner and representative tissue images were exported using NDP.view v2.7.43 software. All figures containing images and graphs were processed using Adobe Illustrator 25.2.1.

### Data Availability

The mass spectrometry proteomics data have been deposited to the ProteomeXchange Consortium via the PRIDE^28^ partner repository with the dataset identifier PXD045268.

## Results

We assembled a case series of 40 embryonal brain tumors (16 ETMR, 9 AT/RT, 15 MB). ETMR diagnoses had been molecularly confirmed through evidence of a C19MC amplification via fluorescence in situ hybridization (FISH) and/or an ETMR-specific epigenetic profile (MNP classifier match with the methylation class “Embryonal tumor with multilayered rosettes” using v11b4 or v12.5 (Supplementary Table 1, Supplementary Figure 1).^29^ Representative FFPE tumor material of all cases was subjected to proteome analyses via liquid chromatography coupled with tandem mass spectrometry (LC–MS/MS).

### ETMR Proteomics Strongly Relate to Histological Subtypes

**Figure 1.**
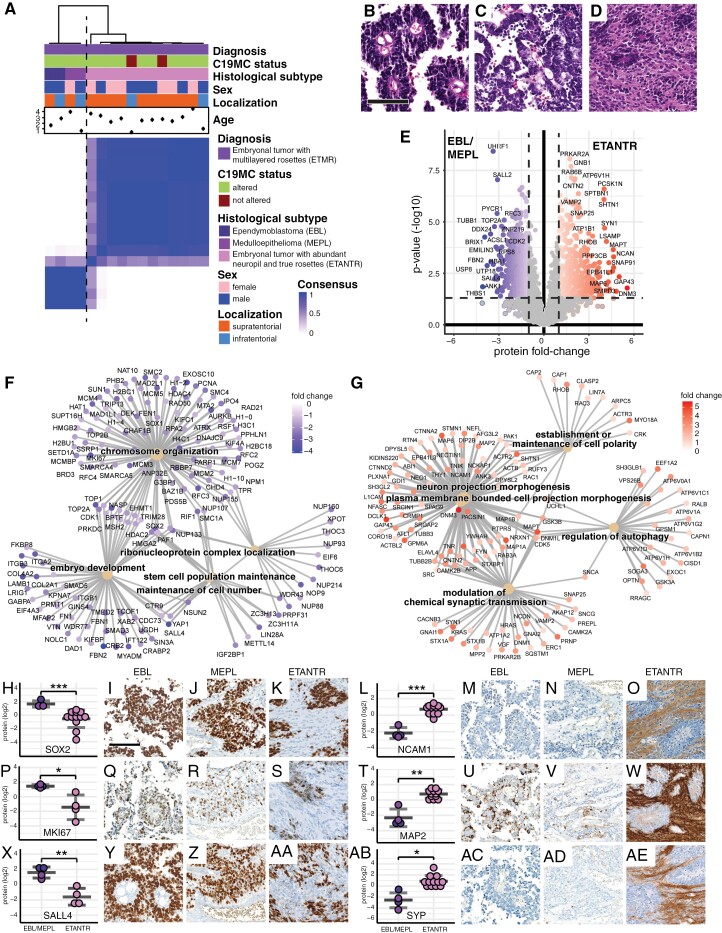
ETMR proteomics are closely associated with histomorphology (A) Consensus clustering of proteome data shows that ETMR samples cluster according to their histological variants. Cases are separated into a proteome subgroup of ependymoblastomas (EBL) and medulloepitheliomas (MEPL) and another proteome subgroup of embryonal tumors with abundant neuropil and true rosettes (ETANTR). Tissue samples were prepared to be representative of the morphology of the entire tumor. Clustering was based on the 1000 most variant proteins, Euclidean distance, and ward.D2 linkage. *K* = 2 is shown. (B)**—**(D) Representative images of ETMR histological variants. EBL shows multilayered rosettes surrounding small lumina (B). MEPL exhibits epithelioid features with columnar or tubular architecture (C). ETANTR are characterized by vast areas of eosinophilic fibrillary matrix and occasional rosettes (D). Scale bar is 100 µm in B—D. **(E)** Volcano plot shows differentially abundant proteins comparing ETANTR to EBL/MEPL tumors. Fold-changes of mean abundancies are shown. Welch’s *t*-test. (F) Gene ontology (GO) category-gene-net (CNET) plot of the top 5 significant results derived from GO overrepresentation analysis (ORA) in all proteins with significantly higher abundance in EBL/MEPL compared to ETANTR. GO subontology: biological process (BP). (G) GO CNET plot of the top 5 significant results of ORA in all proteins with significantly higher abundance in ETANTR compared to EBL/MEPL. GO subontology: BP. (H—AE) Representative protein markers and corresponding immunohistochemical stains demonstrate the influence of neuropil-rich areas on ETMR proteomics. Stemness- and proliferation-associated markers like SOX2 (H-K), MKI67 (KI67, P-S), and SALL4 (X-AA) are strongly expressed in EBL/MEPL and the rosettes of ETANTR. Neuronal markers like NCAM1 (CD56, L-O), MAP2 (MAP2C, T-W), and Synaptophysin (SYP, AB-AE) are predominantly expressed in the neuropil of ETANTR. *P ≤ .05, **P < .01, ***P < 0.001, Welch’s *t*-test. Scale bar is 100 µm in all histological images in H—AE.

First, we aimed to explore the proteome landscape of the ETMR case series. To illustrate similarities and differences between the samples, we employed hierarchical cluster analyses. We found that ETMR separated into 2 distinct and stable proteome subgroups (Figure 1A; Supplementary Figure 2A and B). The first subgroup harbored all tumors, with either EBL or MEPL (Figure 1A–C) morphology, whereas the second subgroup harbored all tumors classified as ETANTR (Figure 1A and D). Of note, the latter group contained 2 ETMR cases without C19MC amplification, which did not specifically separate from C19MC-altered tumors (Figure 1A; Supplementary Figure 2A and B; Supplementary Figure 3A–J). We next investigated the differential proteomics of these 2 subgroups, focusing on the proteins with significantly differential abundance in ETANTR compared to EBL/MEPL cases (Figure 1E; Supplementary Table 2A). Through gene ontology (GO) over-representation analysis (ORA), we found an enrichment of proteins relating to stemness, developmental processes, and chromosome organization in EBL/MEPL cases (Figure 1F; Supplementary Figure 3A; Supplementary Table 2B). In the ETANTR cases, on the other hand, we found an enrichment of proteins relating to neuronal signatures (Figure 1G; Supplementary Figure 3A; Supplementary Table 2C).

We, therefore, hypothesized that the ETMR neuropil portrays the histomorphological correlate of neuronal differentiation, and suspected that the ETMR neuropil directs the proteome subgroup affiliation. Immunohistochemical stainings (Figure 1H–AE) confirmed that tumor cells in areas with undifferentiated, rosette-like, or epithelioid morphology of EBL/MEPL cases, as well as rosette structures of ETANTR cases were immunopositive for stemness- and proliferation-associated markers like SOX2, KI67, and SALL4. In contrast, the neuropil-rich areas of ETANTR, were immunonegative for these markers, but instead stained intensely for neuronal markers like CD56, MAP2C, and synaptophysin.

### Tumor Morphology Overpowers Tumor Identity in ETMR Proteomics

**Figure 2.**
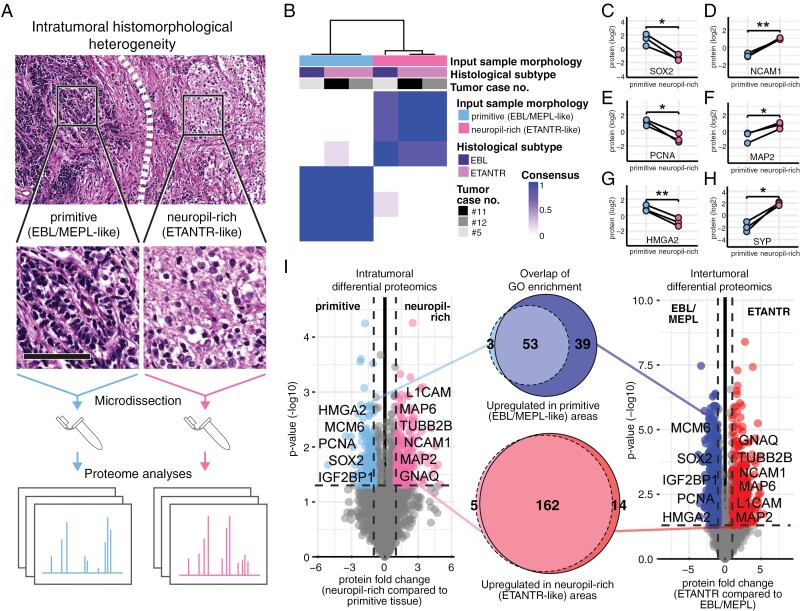
ETMR proteome profiles associate with histomorphological heterogeneity between and within tumors (A) 3 ETMR with demarcated areas of primitive (EBL/MEPL-like) and neuropil-rich (ETANTR-like) morphology were subjected to microdissection with subsequent separate proteome analyses of both tumor areas. Scale bar is 100 µm in insets. (B) Consensus clustering of 3 microdissected tumors shows that microdissected samples cluster according to histomorphology of the tumor area, overpowering the superordinated histological tumor subtype and the patient/tumor identity. Clustering was based on the 1000 most variant proteins, Euclidean distance, and ward.D2 linkage. *K* = 2 is shown. (C—H) Representative proteins demonstrate the presence of proliferation and stemness markers (SOX2 (C), PCNA (E), and HMGA2 (G)) in the primitive areas and the presence of neuronal markers (NCAM1 (D), MAP2 (F), and SYP (H)) in the neuropil-rich areas. *P ≤ .05, **P < 0.01, paired *t*-test. **(i)** Comparison of functional enrichment in intra- vs. intertumoral differential proteomics. Left volcano plot shows the intra-tumorally differentially abundant proteins comparing neuropil-rich (ETANTR-like) with primitive (EBL/MEPL-like) tumor areas (paired *t*-test). Right volcano plot shows the inter-tumorally differentially abundant proteins comparing ETANTR with EBL and MEPL (Welch’s *t*-test). Venn diagrams show the overlap of terms derived from separate ORA of significantly differentially abundant proteins. GO subontology: BP.

Next, we aimed to demonstrate the magnitude of morphology and proteome interconnection in ETMR. Three of the 16 ETMR (cases #5, #11 and #12) were exceptionally heterogeneous and showed vast, well-demarcated areas of either neuropil-rich (ETANTR-like) or primitive (EBL/MEPL-like) architecture. Both areas were microdissected and separately subjected to proteome measurements in all 3 cases (Figure 2A). The consensus clustering showed that the 6 generated samples clustered according to tissue morphology and not according to the tumor’s superordinate histological subtype or the tumor’s identity (Figure 2B; Supplementary Figure 2C and D). We continued to investigate proteins with intratumoral differential abundance in neuropil-rich compared to primitive areas. Stemness- and proliferation-associated markers like SOX2, PCNA, and HMGA2, were found in higher abundance in the primitive areas (Figure 2C, E, G; Supplementary Figure 4B), whereas neuronal markers such as CD56, MAP2C, and synaptophysin, were found in higher abundance in neuropil-rich areas (Figure 2D, F, H; Supplementary Figure 4D). Finally, we found that GO enrichment relating to intratumoral differential proteome profiles was highly analogous to the intertumoral differential proteomics (Figure 2I; Supplementary Table 3A–D). We concluded that the heterogeneity of ETMR proteomics is primarily reflective of histomorphological features, regardless of inter- or intra-tumor comparisons.

### Comparative Proteomics in Embryonal Brain Tumors Reveal Unique Proteome Features of ETMR

**Figure 3.**
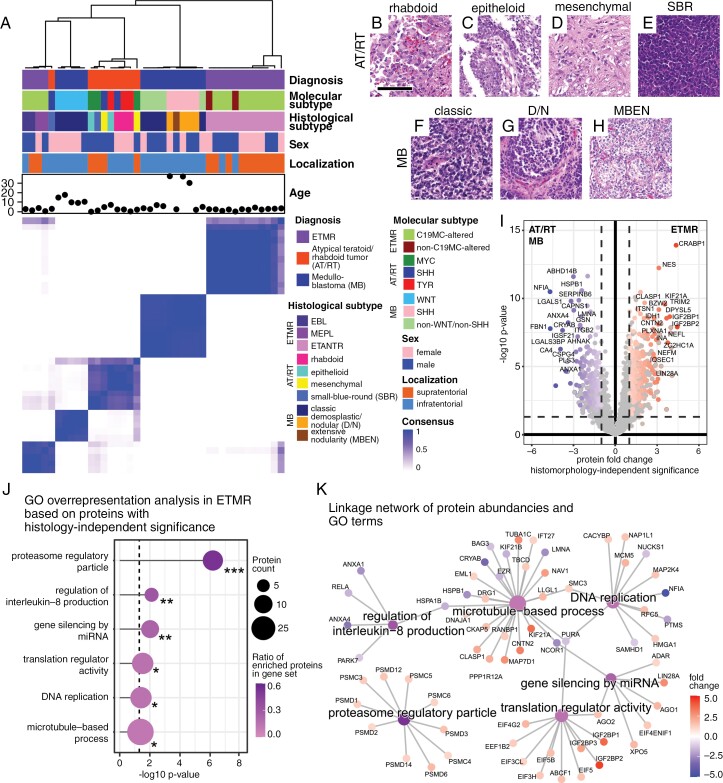
Embryonal brain tumor proteomics showcase distinct features of ETMR (A) Consensus clustering of proteome data shows that ETMR samples cluster separately from other embryonal brain tumors (AT/RT and MB). Of note, histomorphology dictates proteome clustering in ETMR more strongly than in AT/RT and MB. Clustering was based on the 1000 most variant proteins, Euclidean distance, and ward.D2 linkage. *K* = 5 is shown. (B)—(H) Representative images of histological variants of AT/RT and MB. AT/RT tumor morphology was rhabdoid with abundant eosinophilic cytoplasm and eccentrically located nuclei (B), epithelioid with cohesive growth and formation of surfaces (C), mesenchymal with spindled cells and desmoplasia (D) or undifferentiated with basophilic small-blue-round (SBR) appearance of cells (E). MB tumor morphology was classic with densely packed, poorly differentiated cells (F), desmoplastic/nodular (D/N) with islands of tumor cells separated by connective tissue fibers (G) or extensively nodular (MBEN, H). Scale bar is 100 µm in B—H. (I) Volcano plot shows differentially abundant proteins of ETMR compared to AT/RT and MB. Coloring indicates ETMR histomorphology-independent significant differential abundance. Significance was considered histomorphology-independent, when proteins showed a |fc| > 1 and *P*-value ≤ .05 in ETANTR as well as EBL/MEPL compared to AT/RT and MB. Welch’s *t*-test. (J) Lollipop plot shows representative GO terms associated with differentially abundant proteins in ETMR compared to AT/RT and MB. The shown terms were significant and morphology-independent in both ORA and GSEA. ORA statistics are shown. GO subontology: BP, molecular function (MF) and cellular component (CC). (K) CNET plot shows the linkage of significant GO terms, proteins, and protein fold changes comparing ETMR with AT/RT and MB. Of note “proteasome regulatory particle” proteins are consistently higher abundant in ETMR compared to AT/RT and MB.

We continued to compare ETMR proteome signatures to those of other malignant pediatric embryonal brain tumors (9 AT/RT and 15 MB; Supplementary Table 1). Consensus clustering of embryonal brain tumors showed that ETMR proteome signatures are distinct from AT/RT and MB (Figure 3A). Notably, histomorphological subtypes of AT/RT^30^ and MB^31^ did not associate with tumor entity subclusters nearly as evidently as in ETMR (Figure 3A–H; Supplementary Figure 2E and F). We next aimed to further investigate unique proteome features of ETMR in search of potential therapeutic vulnerabilities.

Intratumoral heterogeneity poses a persistent challenge in cancer therapy, as it can foster tumor evolution under the selective pressure of medical treatments.^32^ Given the close association of histomorphology and proteome profiles we found between, but also within ETMR, we inferred that proteome features attributable to both proteome subgroups constitute the most promising prospect for novel therapeutic approaches. To identify intratumorally pervasive proteome features of ETMR, we focused our analyses on proteins with histomorphology-independent significantly differential abundance in ETMR compared to AT/RT and MB (Figure 3I; Supplementary Table 4A–C). Of note, the RNA binding protein LIN28A, which is considered a characteristic immunohistochemical marker in ETMR, was found among the significant candidate proteins (fold change = 2.84 and ***P < .001; Supplementary Table 4A). Significant proteins were subjected to ORA (Supplementary Table 4B) and, additionally, gene set enrichment analyses (GSEA) were performed (Supplementary Table 4C). We identified the terms “proteasome regulatory particle”, “regulation of interleukin-8 production”, “gene silencing by miRNA”, “translation regulator activity”, “DNA replication”, and “microtubule-based process” to be representative of the significantly enriched GO terms in both analyses (Figure 3J, Supplementary Table 4B and C). Notably, “proteasome regulatory particle” was the most significant gene set hit (***P < .001) and exclusively comprised gene set members with higher protein abundance in ETMR independent of histomorphology (Figure 3K). The prominence of these gene sets as histomorphology-independent features of ETMR was confirmed by separate ORA of EBL/MEPL and ETANTR (Supplementary Figure 5A and B). The QIAGEN Ingenuity Pathway Analysis (IPA) platform was used to search for potential therapeutic targets in the ETMR proteome.^27^ A search based on proteins, which were highly abundant in ETMR independent of histomorphology revealed proteasome inhibition as a potential target (Supplementary Table 5A). Moreover, analysis of EBL/MEPL-specific proteins pointed out TOP2A inhibitors such as anthracyclins (Supplementary Table 5B). Abundance of the synaptic protein SV2A in ETANTR associated with antiepileptic anticancer drugs like levetiracetam (Supplementary Table 5C).

### Integrated Proteomics of Embryonal Brain Tumors Decipher Highly and Poorly Epigenetically Conserved Pathways in ETMR

**Figure 4.**
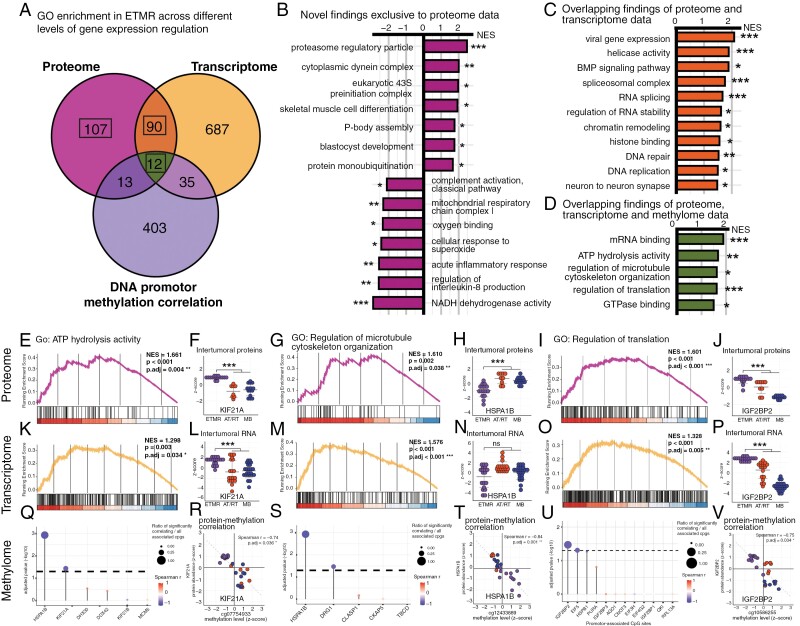
Integrated proteomics add new insights to ETMR molecular biology and reflect transcriptome-based features (A) Venn diagram demonstrates the quantitative overlap of GO term enrichment associated with the proteome, transcriptome, and methylome of ETMR. GO enrichment in the proteome and transcriptome was determined via GSEA comparing ETMR to AT/RT and MB. GO enrichment in the methylome was determined via GO over-representation analysis of proteins showing a significant correlation with their promotor methylation levels. (B—D) Bar graphs show selected GO terms, which were either associated with molecular signatures reflected in the ETMR proteome exclusively (B), with signatures reflected in the ETMR proteome and transcriptome (C), or with signatures reflected in the ETMR proteome, transcriptome as well as the methylome (D). Protein level GSEA statistics in ETMR are shown. NES: normalized enrichment score. BH-adjusted *P*-values: *P ≤ .05, **P < .01, ***P < 0.001. GO subontology: BP, MF and CC. (E—V) Enrichment analyses results are shown for 3 selected GO terms and their association with the ETMR proteome (E—J), transcriptome (K—P), and methylome (Q—V): “ATP hydrolysis activity” (E, K, Q), “regulation of microtubule organization” (G, M, S) and “regulation of translation” (I, O, U). Protein and RNA levels as well as correlation plots of proteins and promotor methylation levels are representatively shown for the proteins KIF21A (F, L, R), HSPB1A (H, N, T), and IGF2BP2 (J, P, V). All *P*-values derived from GSEA were BH-adjusted. *P ≤ .05, **P < .01, ***P < .001.

To further explore ETMR tumor biology, we acquired transcriptome data of ETMR, AT/RT, and MB from publicly available datasets (Supplementary Table 6).^6,14–16^ Consensus clustering of the compiled data confirmed robust clustering of tumor entities based on transcriptome profiles (Supplementary Figure 6A), as previously described.^16^ Tissue from a subset of embryonal brain tumors of our case series was also available for global DNA methylation analysis (*n* = 28). As expected, and previously described,^29^ consensus clustering based on methylome data showcased robust clustering of samples according to tumor entities (Supplementary Figure 6B). We proceeded to explore GO term enrichment in ETMR via GSEA on the 3 different *omic* datasets, reflecting steps of the central dogma of gene expression (ie protein, RNA, and DNA promoter methylation). Subsequently, we searched for overlap of GO terms separately detected in all 3 analyses (Figure 4A, Supplementary Table 7).

We identified 107 GO terms of gene sets enriched in ETMR exclusively in the proteome. Among the significantly positively enriched terms was again the “proteasome regulatory particle.” Among the significantly negatively enriched terms were, among others, the “mitochondrial respiratory chain complex I”, “oxygen binding”, and “NADH dehydrogenase activity” (Figure 4B; Supplementary Table 7A).

We identified 90 GO terms of gene sets enriched in both the proteome and transcriptome of ETMR. Among the significantly positively enriched terms we identified, as expected from previous work,^2,6,33^ “helicase activity”, “RNA splicing”, “chromatin remodeling”, “histone binding” and “DNA repair” (Figure 4C; Supplementary Table 7B).

GSEA results generated by separately investigating EBL/MEPL and ETANTR confirmed that the aforementioned GO terms were attributable to ETMR independent of histomorphology (Supplementary Figure 5C).

As epigenetically conserved features, we identified 12 GO terms with overlapping representation in the proteome, transcriptome as well as the methylome of ETMR. We determined “mRNA binding”, “ATP hydrolysis activity”, “regulation of microtubule cytoskeleton organization”, “regulation of translation”, and “GTPase binding” to best portray these terms (Figure 4D; Supplementary Table 7C). Among the protein members of these gene sets, HNRNPAB, IFG2BP2, HSPA1B, KIF21A, ANXA2, and NCKAP1 showed significant correlation with their respective genes’ promotor methylation levels (Supplementary Table 7E). Of note, KIF21A and IGF2BP2 were also among the proteins we identified to simultaneously display significantly higher protein abundance, higher RNA levels, and lower promotor methylation levels in ETMR (Figure 4E–V, Supplementary Figure 7). Our results suggest these genes to be highly epigenetically conserved in ETMR.

### Proteasome Regulatory Protein Abundancy Is a Unique Molecular Characteristic of ETMR

**Figure 5.**
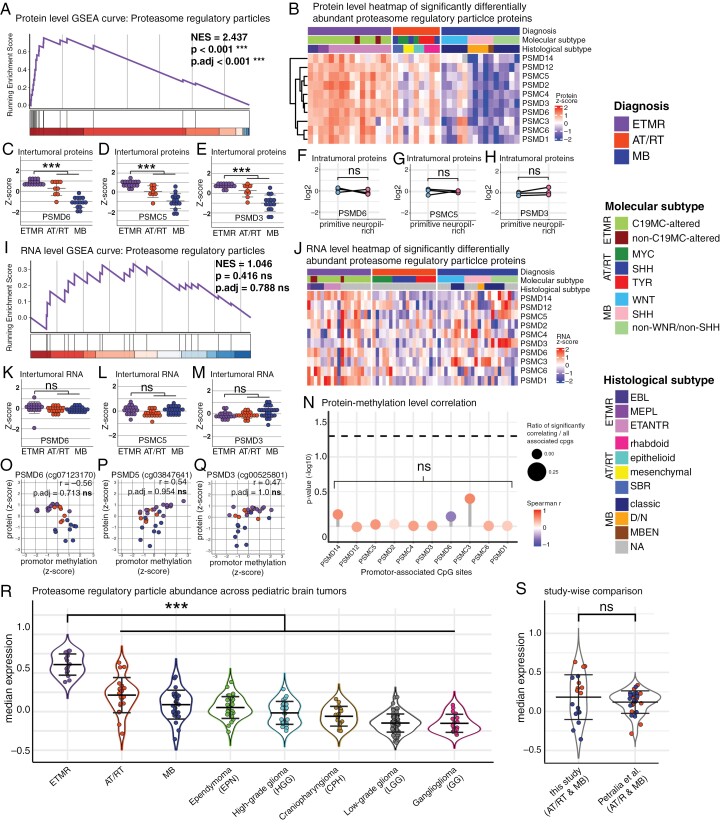
Proteasome regulatory particle proteins are highly abundant in ETMR (A) GSEA curve of the “proteasome regulatory particle” gene set based on protein abundancies comparing ETMR to AT/RT and MB. *P*-value was BH-adjusted. (B) Proteome heatmap shows a pervasive abundance of proteasome regulatory particles in ETMR compared to AT/RT and MB. (C—E) Plots show protein levels of the 3 top ranked proteasome regulatory particle gene set members PSMD6 (C), PSMC5 (D), and PSMD3 (E). ***P < .001. Welch’s *t*-test. (F—H) Microdissection analyses confirm that protein abundance of PSMD6 (F), PSMC5 (G), and PSMD3 (H) is not associated with histomorphology of ETMR. (I) GSEA curve of the “proteasome regulatory particle” gene set based on RNA levels comparing ETMR to AT/RT and MB shows no significant enrichment for ETMR. *P*-value was BH-adjusted. (J) Transcriptome heatmap of “proteasome regulatory particle” RNA levels appears noisy across embryonal brain tumors. (K—M) Plots show RNA levels of the 3 top ranked proteasome regulatory particle gene set members PSMD6 (K), PSMC5 (L), and PSMD3 (M). No significant fold changes can be seen. *P*-values were BH-adjusted. ns: not significant. Welch’s *t*-test. (N) Lollipop plot shows the correlation of protein and promotor methylation levels of “proteasome regulatory particle” gene set members. No members show a significant correlation with any CpG sites associated with their respective promotor regions. Spearman correlation. *P*-values were BY-adjusted. ns = not significant. (O—Q) Plots show protein levels and the methylation levels of the representative promotor CpG sites across embryonal brain tumors. No significant correlation is seen in the 3 top ranked proteasome regulatory particle gene set members PSMD6 (O), PSMC5 (P), and PSMD3 (Q). (R, S) Integrated proteome data of our study and publicly available proteome data of further pediatric brain tumors (Petralia et al.) demonstrates that the abundancy of proteasome regulatory particles is a prominent molecular feature of ETMR. Median protein abundance of all “proteasome regulatory particle” gene set members are shown across entities (R) and comparing overlapping entities (AT/RT and MB) represented in both datasets (S). The latter plot confirms that differential protein levels do not relate to batch effects. ***P < .001. Welch’s test.

We continued to focus our investigations on the abundance of proteasome regulatory particle proteins in ETMR. This finding was of particular interest since it represented a highly significant novel molecular feature of ETMR that did not associate with intratumoral histomorphology-based heterogeneity and was not reflected on preceding levels of gene expression regulation, i.e. RNA or DNA methylation (Figure 5A–Q). We integrated our proteome data set of embryonal brain tumors with publicly available protein data of other pediatric brain tumors, comprising the tumor entities ependymoma, craniopharyngioma, ganglioglioma, high- and low-grade glioma as well as further AT/RT and MB cases.^21^ Comparisons across pediatric brain tumor entities demonstrated that proteasome regulatory protein abundance was the highest in ETMR (***P < .001), underlining the distinctiveness of this molecular feature (Figure 5R and S).

### Proteasome Inhibition Poses a Promising Therapeutic Approach in ETMR

**Figure 6.**
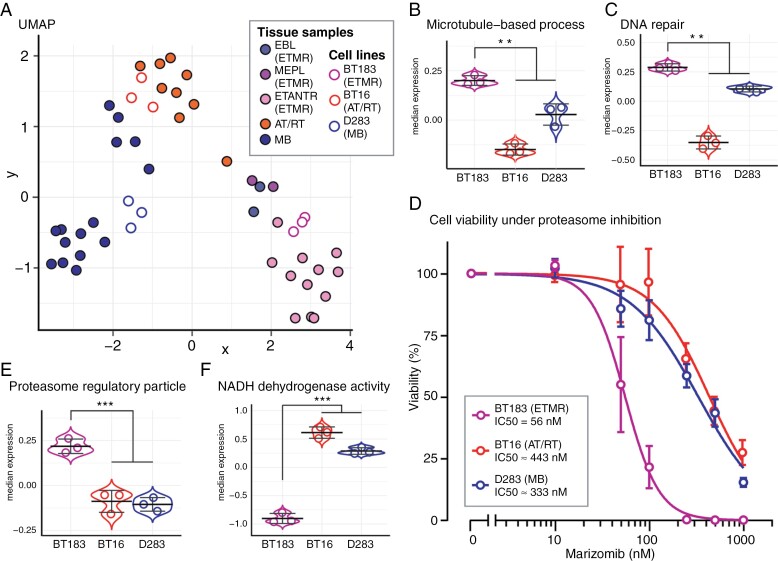
Proteasome inhibition represents a promising therapeutic vulnerability in ETMR (A) UMAP plot shows that the proteome profiles of the cell lines BT183, BT16, and D283 adequately match with ETMR, AT/RT, and MB tumor tissue, respectively. UMAP dimension reduction was based on the top 1000 most significant proteins of an ANOVA test between ETMR, AT/RT and MB tissue samples. (B—C) Median protein abundance of all “Microtubule-based process” and “DNA repair” gene set members are shown in BT183, BT16 and D283. Proteins of both gene sets are significantly more abundant in BT183 compared to BT16 and D283. **P < .01. Welch’s *t*-test. (D) Cell titer glo viability assay of BT183 (ETMR), BT16 (AT/RT), and D283 (MB) cell lines treated with the CNS penetrant proteasome inhibitor Marizomib at different concentrations. BT183 ETMR cells are highly vulnerable towards Marizomib treatment, also in comparison with other embryonal tumor cell lines. Four parameter logistic (4PL) curve regression model. (E—F) Median protein abundance of all “Proteasome regulatory particles” and “NADH dehydrogenase activity” gene set members are shown in BT183, BT16, and D283. Proteasome regulatory particle proteins are highly abundant in BT183 compared to BT16 and D283. In contrast, NADH dehydrogenase activity proteins are significantly less abundant in BT183 compared to BT16 and D283. ***P < 0.001. Welch’s *t*-test.

We hypothesized that the abundance of proteasome regulatory proteins reflects a strong dependency of ETMR on the proteasome to safeguard proteostasis. To prove this concept in vitro, we proceeded to investigate the embryonal brain tumor cell line BT183, which was derived from a C19MC-amplified ETMR with large areas of neuropil in the posterior fossa of a 2-year-old boy.^34^ Proteome data were generated from BT183, as well as the AT/RT cell line BT16, and the MB cell line D283. The proteome profiles of these cell lines adequately matched with their respective tumor entity (Figure 6A). Of note, the BT183 proteome profile best matched with ETANTR profiles (Supplementary Figure 8A–E) and, in analogy to the results derived from ETMR tissue samples, showed ETMR-characteristic significant abundance of proteins associated with microtubule-based processes and DNA repair (Figure 3K; Figure 4C, D; Figure 6B and C). Moreover, as in the tissue samples, proteasome regulatory particles were highly abundant and NADH dehydrogenase activity proteins were significantly less abundant in BT183 compared to BT16 and D283 (Figure 4B; Figure 5A–E; Figure 6E and F). We therefore concluded that these cell lines were suitable to test the potential therapeutic vulnerability of ETMR against proteasome inhibition and proceeded to perform in vitro experiments. All cell lines were treated with the CNS-penetrant proteasome inhibitor Marizomib and assayed for cell viability. We found that BT183 was exceptionally vulnerable toward treatment with Marizomib (IC50 = 56 nM) compared to BT16 and D283 (IC50s ≈ 443 nM, and 333 nM, respectively; Figure 6D). In summary, we demonstrated that proteasome regulatory particle abundance is a distinctive, histology-independent feature of ETMR and that proteasome inhibition represents a promising therapeutic vulnerability in ETMR.

## Discussion

The main ETMR-related novel findings of this report comprise (i) the close association of histomorphology and proteome signatures, (ii) an outline of tumor-specific molecular features depicted on different levels of gene expression regulation, (iii) an abundance of proteasome regulatory proteins, and (iv) the detection of proteasome inhibition as a promising therapeutic vulnerability. As embryonal brain tumors, especially ETMR, are quite rare, our study is limited by rather small case numbers. Consequently, not all molecular subtypes may be sufficiently represented in our series. For example, our series comprised only 2 non-C19MC-altered ETMR.

Based on transcriptome data, Lambo et al. previously linked miRNA-related aberrations to R-loop-associated chromosomal instability with high DNA repair expression and helicase activity in ETMR.^6^ As we also identified RNA processing mechanisms, gene silencing by miRNA, DNA repair, and helicase activity to be reflected on the proteome level, our study confirms these molecular features of ETMR. Roles of Hippo, NOTCH, WNT, and SHH signaling have also been described in ETMR.^6,35^ Although several respective relevant gene set members were identified in our data, we could not confirm a significant enrichment of these pathways on the proteome level. However, statistical analyses revealed tendencies of enrichment, therefore these results might change with higher case numbers. Also, the reference samples used in this study may have affected the analytical results, since several subtypes of embryonal tumors rely on congruent pathway activations (e.g. SHH- and WNT-signaling in MB-SHH and MB-WNT, respectively). A recent study suggested that *C19MC* and LIN28A drive ETMR progression via a MYCN-mediated core transcriptional regulatory circuitry involving super-enhancer transcription factors.^36^ Transcription factors are generally expressed with low abundancies and therefore, their detection is limited in proteome analyses.^37^ Accordingly, major members of this transcriptional regulatory network such as MYCN, MAZ, SOX11, SALL1, and SP3^36^ were not detected in our analyses.

We show that proteome signatures of ETMR are closely associated with histomorphological features. Previous studies have suggested a similar association between the ETMR transcriptome and histomorphology. In detail, transcriptional signatures related to DNA repair mechanisms were reported in ETMR rosettes whereas transcripts of astroglial differentiation were found in ETMR neuropil.^6^ Although neuropil was characterized by a neuronal rather than an astroglial signature in our study, we generally confirm the close linkage of ETMR histomorphology and gene expression features. Importantly, distinctive markers of intratumoral heterogeneity showed similar expression patterns in the previously published transcriptome analyses of microdissected ETMR regions and our proteome analyses.

Notably, transcriptomic subgroups relating to histomorphology have not been described in ETMR. In line with this, we detected no association of ETMR histological subtypes and clustering based on transcriptome profiles upon reviewing the annotations of the public data deposits used in this study. This may be because the entire tumor gives name to its histological variant, and analyzed specimens may not have been purposely compiled to representatively portray the histomorphology of the entire tumor. Moreover, the histological subtyping of ETMR may be subject to interobserver variability.

The question arises as to whether the association of morphology and proteome signatures has clinical implications in ETMR. Previous studies demonstrated that overall survival times do not significantly differ between ETMR histological variants.^5^ On the other hand, multiple reports have indicated a linkage between long-term survival and neuronal differentiation in some ETMR.^5,38–40^ Thus, higher case numbers are needed to clarify if and how neuronal or histomorphology-associated proteome signatures may relate to better survival chances in ETMR. Moreover, differential proteomics associated with histomorphological variations between and within ETMR may warrant histology-oriented therapeutic approaches. For example, prevailingly undifferentiated tumors with EBL/MEPL-like morphology harbored DNA topoisomerases in abundance and may thus be more vulnerable towards treatment with anthracyclins.^41^ On the other hand, neuropil-rich tumors showed upregulated synaptic signaling, which implies that antiepileptic drugs like levetiracetam may be beneficial as adjuvant cancer therapeutics in ETANTR and even exhibit antitumoral effects.^42^

Since histomorphology-associated intratumoral proteome heterogeneity may facilitate tumor evolution under therapeutic selection pressure, we focused on pervasive proteome features of ETMR in this study. Among these features, we discovered understudied molecular characteristics of ETMR, which were not reflected in previously investigated levels of gene expression. For example, we discovered a low abundance of proteins involved in mitochondrial respiration in ETMR. Mitochondrial metabolism has been implicated in multi-functional roles in malignant tumor progression^43,44^ and also is linked to sensitivity towards proteasome inhibition in cancers.^45,46^ The roles of mitochondria for tumorigenesis and progression in ETMR are widely unknown.^47^

The proteasome is an essential multi-subunit complex, which maintains proteostasis by degrading misfolded or damaged proteins.^48,49^ Proteasome regulatory particles regulate the entry and delivery of proteins to the complex’s catalytic core.^50^ Previous studies have linked high levels of proteasome regulatory particles to cytotoxic sensitivity towards proteasome inhibition.^45,51^ Proteasome regulatory particles were highly abundant in ETMR and BT183 cells, while in vitro assays demonstrated the vulnerability of ETMR cells toward proteasome inhibition using Marizomib. In line with our findings, a previous drug screen in BT183 tumor cells identified the proteasome-inhibitor Bortezomib as a potent in vitro ETMR drug candidate.^52^ However, since Bortezomib does not cross the blood–brain barrier, its clinical potential for ETMR treatment was not further pursued in vivo.^52,53^ In recent years, the CNS penetrant proteasome-inhibitor Marizomib has gained attention in the treatment of hematopoietic and solid tumor models.^54,55^ A previous drug screen by Lin et al.^56^ identified the potential of Marizomib to treat cell culture models of diffuse midline glioma (DMG), H3K27 altered. Subsequently, a Phase I study was initiated to investigate Marizomib safety and preliminary efficacy in children with DMG (NCT04341311). A recently published report of this study proclaimed that children tolerated starting doses of Marizomib (0.6 mg/m²) without any dose-limiting toxicities or CNS toxicity.^57^ Notably, Marizomib IC50s of the 6 DMG cell lines used in the study of Lin et al.^56^ were in the range of 10—100 nM and therefore comparable to BT183. Taken together, these results substantiate the therapeutic potential of proteasome inhibition and provide a rationale for future clinical trials to employ Marizomib in ETMR therapy.

## Supplementary Material

noad265_suppl_Supplementary_Figure_S1

noad265_suppl_Supplementary_Figure_S2

noad265_suppl_Supplementary_Figure_S3

noad265_suppl_Supplementary_Figure_S4

noad265_suppl_Supplementary_Figure_S5

noad265_suppl_Supplementary_Figure_S6

noad265_suppl_Supplementary_Figure_S7

noad265_suppl_Supplementary_Figure_S8

noad265_suppl_Supplementary_Table_S1

noad265_suppl_Supplementary_Table_S2

noad265_suppl_Supplementary_Table_S3

noad265_suppl_Supplementary_Table_S4

noad265_suppl_Supplementary_Table_S5

noad265_suppl_Supplementary_Table_S6

noad265_suppl_Supplementary_Table_S7
