# Supplementary figures and images for: Integrated proteomics spotlight the proteasome as a therapeutic vulnerability in embryonal tumors with multilayered rosettes
Source: Neuro Oncol. 2023 Dec 30;26(5):935–49. doi: 10.1093/neuonc/noad265 (PMC11066909; doi:10.1093/neuonc/noad265)

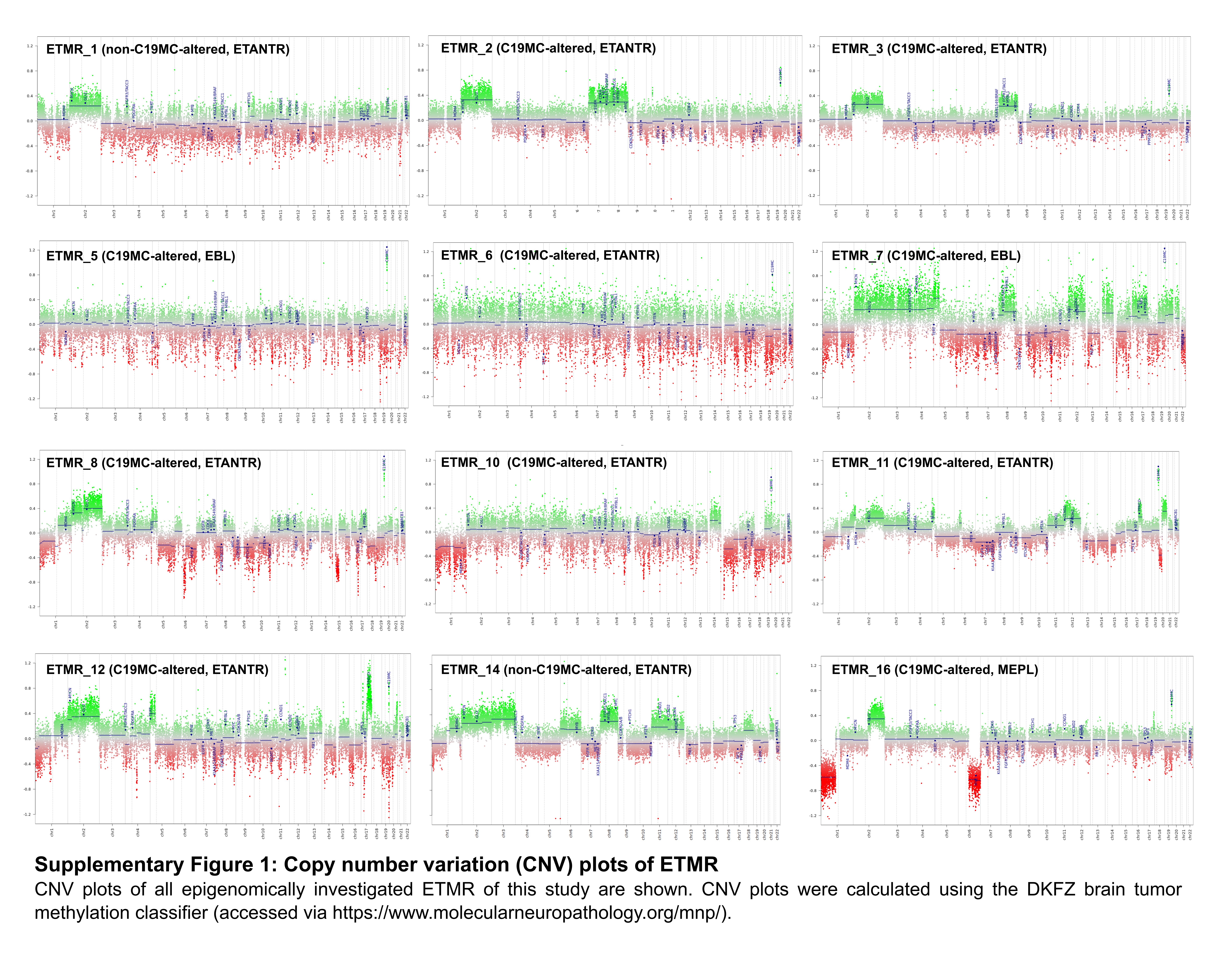

Supplement: noad265_suppl_Supplementary_Figure_S1 [file noad265_suppl_supplementary_figure_s1.jpeg]

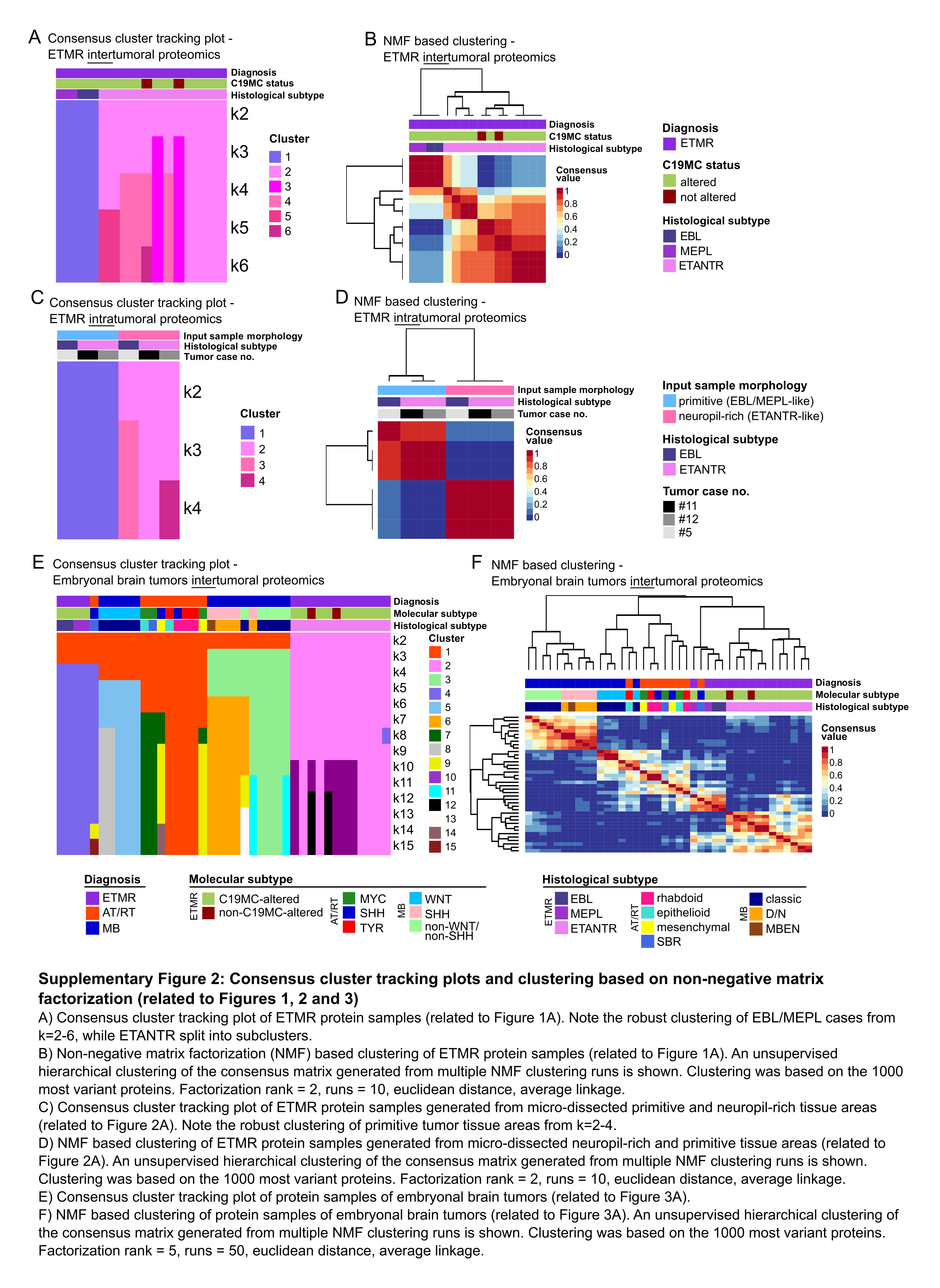

Supplement: noad265_suppl_Supplementary_Figure_S2 [file noad265_suppl_supplementary_figure_s2.jpeg]

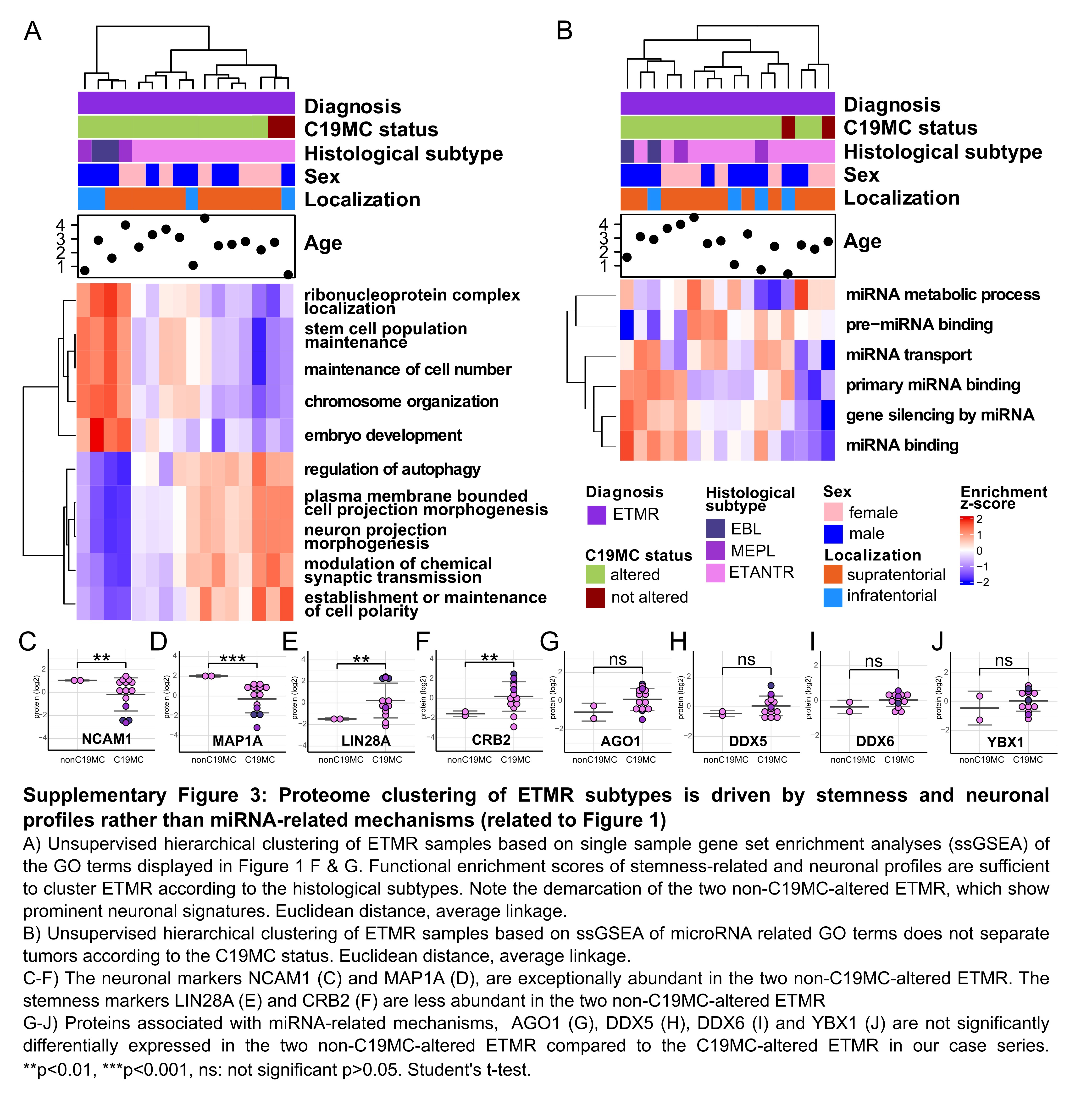

Supplement: noad265_suppl_Supplementary_Figure_S3 [file noad265_suppl_supplementary_figure_s3.jpeg]

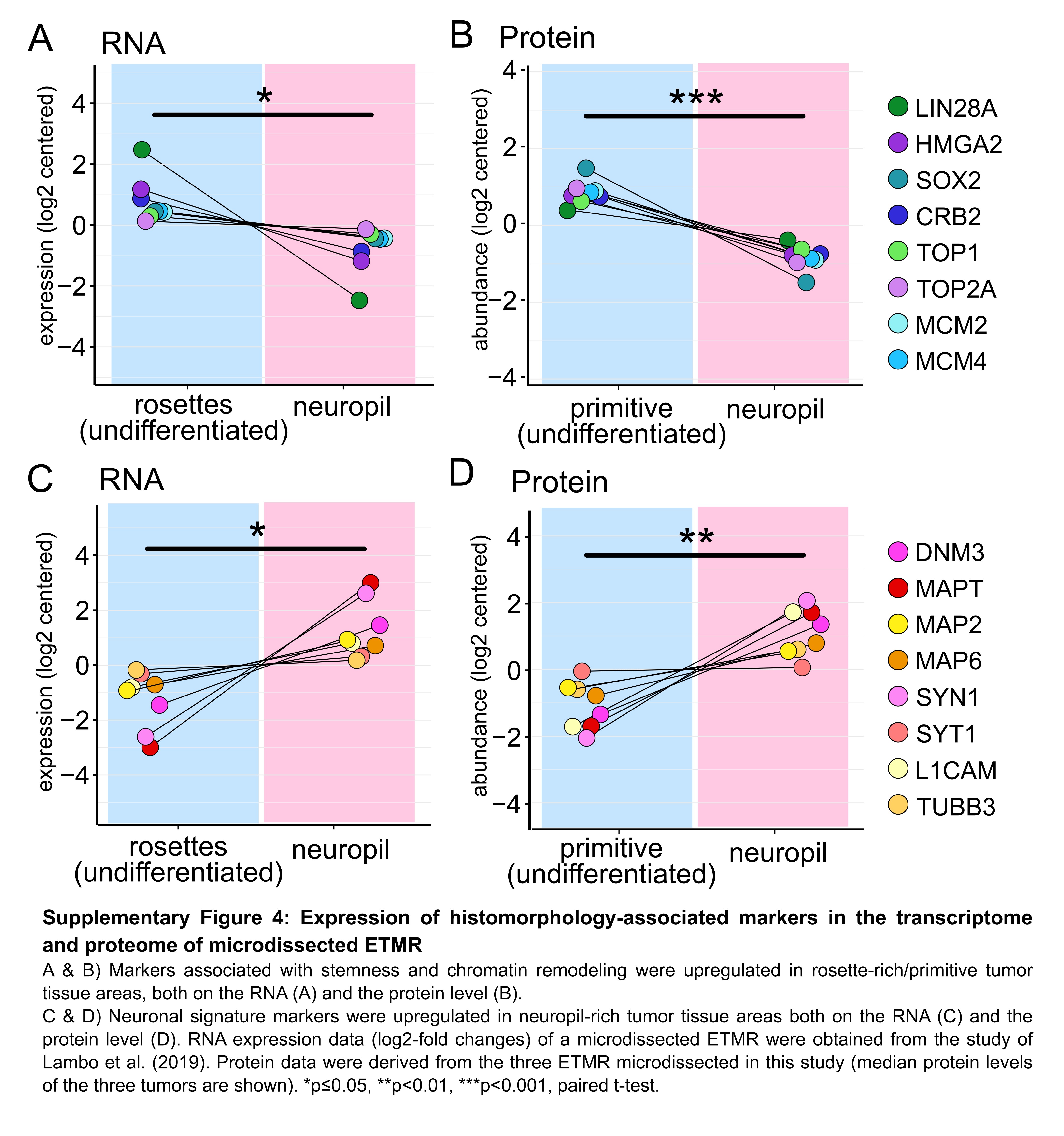

Supplement: noad265_suppl_Supplementary_Figure_S4 [file noad265_suppl_supplementary_figure_s4.jpeg]

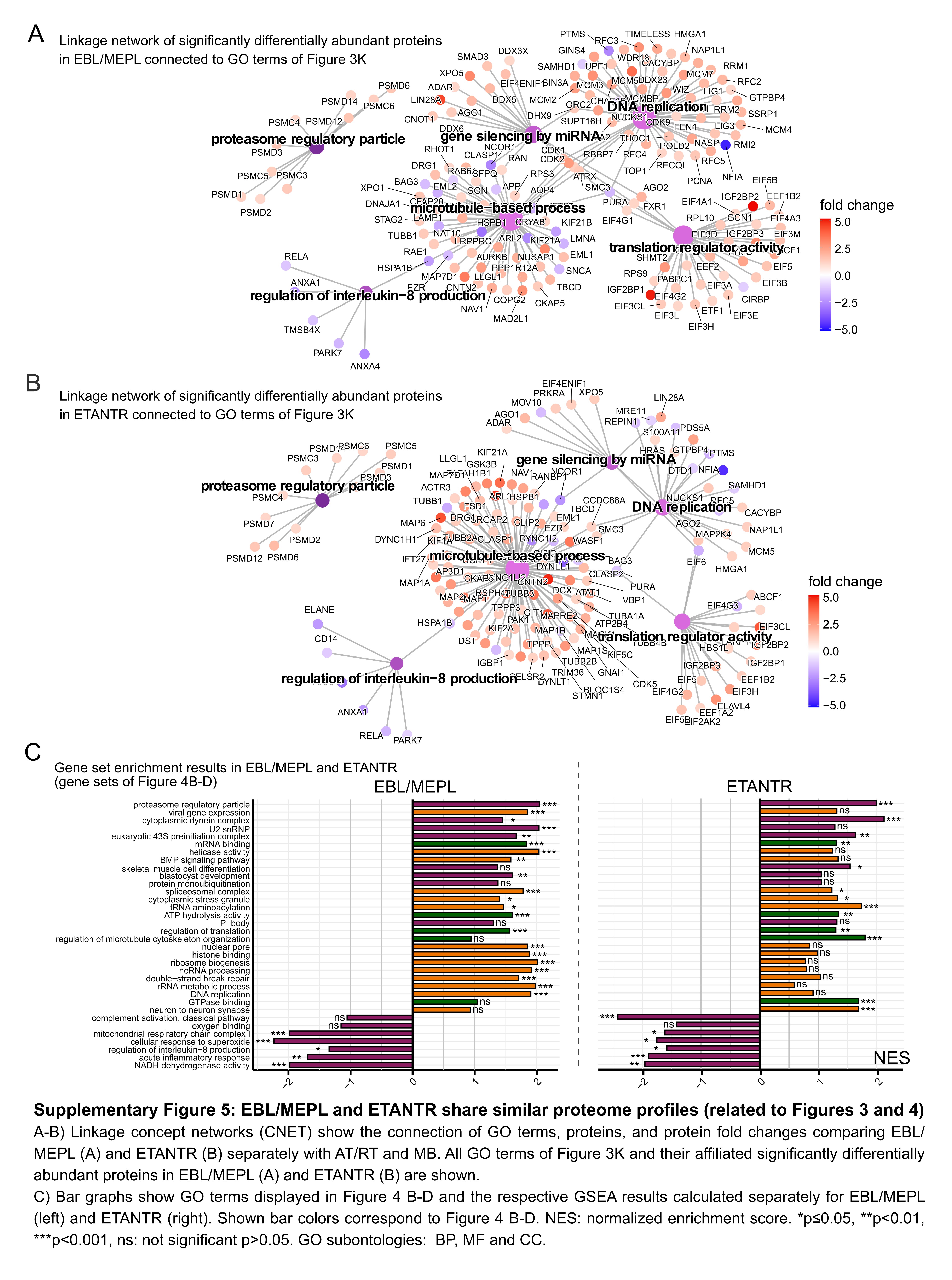

Supplement: noad265_suppl_Supplementary_Figure_S5 [file noad265_suppl_supplementary_figure_s5.jpeg]

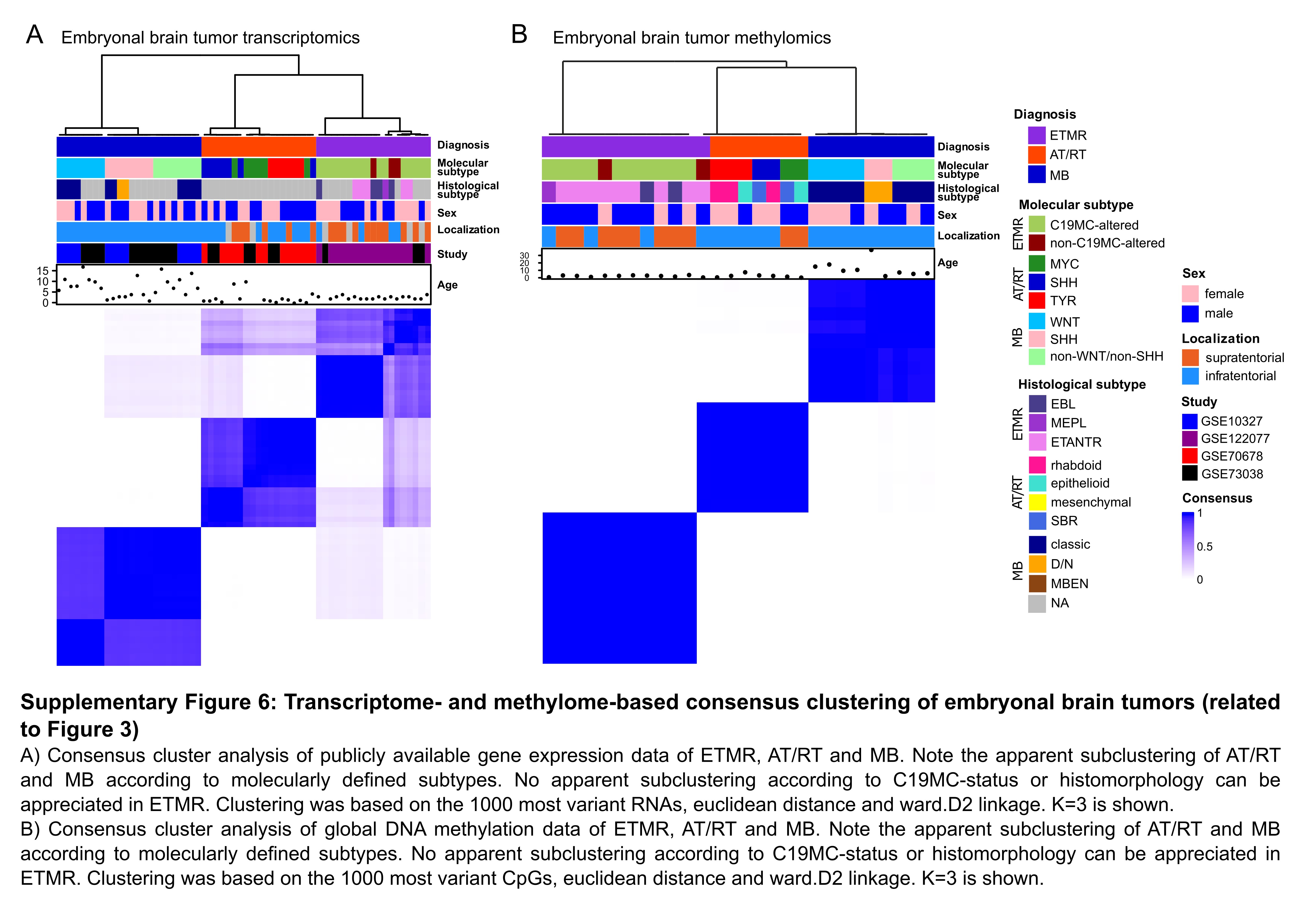

Supplement: noad265_suppl_Supplementary_Figure_S6 [file noad265_suppl_supplementary_figure_s6.jpeg]

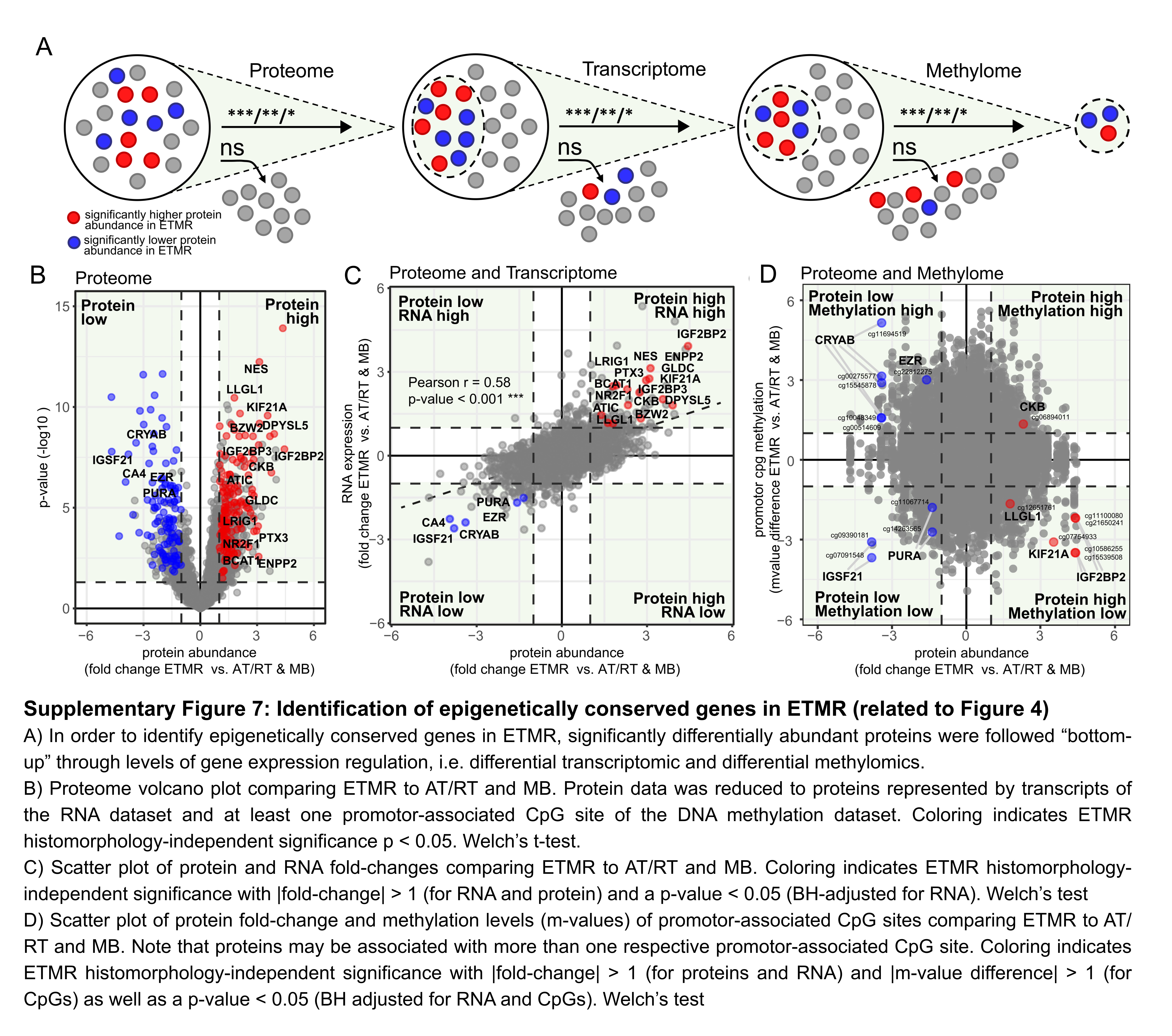

Supplement: noad265_suppl_Supplementary_Figure_S7 [file noad265_suppl_supplementary_figure_s7.jpeg]

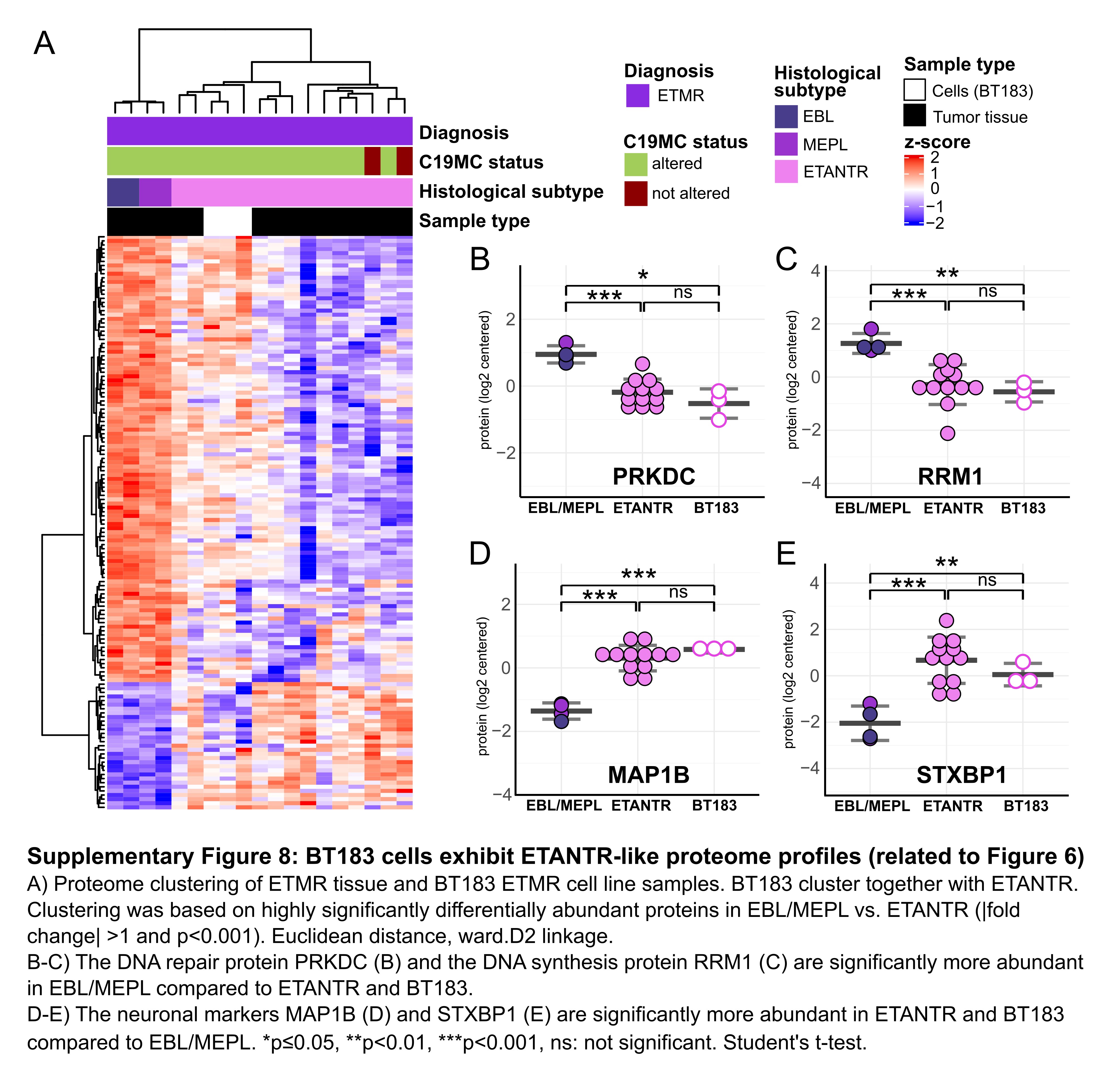

Supplement: noad265_suppl_Supplementary_Figure_S8 [file noad265_suppl_supplementary_figure_s8.jpeg]
